# Safety and Feasibility of Microwave Ablation for Hepatocellular Carcinomas in the Elderly: A Systematic Review

**DOI:** 10.3389/fonc.2022.855909

**Published:** 2022-05-23

**Authors:** Weiren Liang, Weiyuan Hao, Guoliang Shao, Jiaping Zheng, Hui Zeng, Danping Zhou, Hefeng Yao

**Affiliations:** ^1^ Department of Interventional Therapy, The Cancer Hospital of the University of Chinese Academy of Sciences (Zhejiang Cancer Hospital), Institute of Basic Medicine and Cancer (IBMC), Chinese Academy of Sciences, Hangzhou, China; ^2^ Department of Endoscopy, The Cancer Hospital of the University of Chinese Academy of Sciences (Zhejiang Cancer Hospital), Institute of Basic Medicine and Cancer (IBMC), Chinese Academy of Sciences, Hangzhou, China; ^3^ Department of Medical Oncology, Huzhou Traditional Chinese Medicine Hospital of Zhejiang Traditional Chinese Medical University, Huzhou, China

**Keywords:** frequency ablation, hepatocellular carcinoma, microwave ablation, elderly, prognosis, review, treatment

## Abstract

**Background:**

Microwave ablation (MWA) for hepatocellular carcinomas (HCCs) in the elderly has been the subject of new research in recent years. However, there are currently no strong lines of evidence for the prognosis following MWA treatment for HCC in the elderly. Therefore, we conducted a systematic review to assess the safety and feasibility of MWA for HCC in elderly patients.

**Methods:**

Up until August 15, 2021, a comprehensive literature search was undertaken in PubMed, Scopus, CENTRAL (Cochrane Central Register of Controlled Trials), and Google Scholar databases for all published articles. Observational studies reporting the safety and feasibility of MWA for HCC in elderly patients were included. The Newcastle–Ottawa Scale (NOS) was used to measure the quality assessment.

**Results:**

Our review, composed of 7 observational studies, including a total of 7,683 HCC patients, looked at the safety and feasibility of MWA for HCC in the elderly. Current lines of evidence on the risks and outcomes of MWA of HCC treatments in elderly patients are discussed.

**Conclusions:**

According to our findings, elderly patients, even those with a high comorbidity index, benefited from MWA of HCC similar to younger patients. More clinical data are needed to determine selection criteria for elderly HCC patients to increase the possibility of receiving MWA as a potential lifesaving option. As such, further studies evaluating the outcomes of MWA for HCC treatment modalities in elderly patients are warranted.

**Systematic Review Registration:**

https://www.crd.york.ac.uk/prospero/, identifier CRD42021273091.

## Introduction

Hepatocellular carcinoma (HCC), the most prevalent primary liver cancer, is the world’s fifth most common cancer and the third leading cause of cancer-related death (1). As life expectancy has increased, the number of older people with HCC has also increased (2). It is widely acknowledged that aging is a risk factor for HCC development (3). Recent studies from the United States, the United Kingdom, and Japan have found a significant age-related rise in the development of HCC in those over the age of 75 (4). In the elderly aged >71 years, it has been found that liver weight (5) and portal blood flow velocity decrease (6), resulting in a reduced liver repair capability compared to younger people. As a result, elderly individuals with liver cancer can expect a worse prognosis after treatment. As human life expectancy increases, a number of studies have advised that the minimum age for elderly groups should be 75 years (7, 8). The majority of studies have found that age distribution at HCC diagnosis has shifted over time, and that those 65 and older with HCC had fewer effective treatments and worse prognoses than younger adults (2, 9, 10).

Management of malignant diseases in elderly individuals is becoming a prominent concern worldwide as the population ages due to improved treatment and healthcare (11). For the majority of older persons, surgery or liver transplantation is difficult (12). As a result, novel therapeutic techniques for the treatment of HCC, such as local radical ablation, targeted chemotherapeutic drugs, and radiation therapy, continue to be researched and developed (13). Hepatic resection (HR) is generally considered as the first-line treatment for HCC patients (10). Studies have shown the feasibility and safety of HR for elderly patients with HCC. The indication of HR is limited because of comorbidity or a poor general status of elderly patients (10). Radiofrequency ablation (RFA), trans-arterial chemoembolization (TACE), and microwave ablation (MWA) have received recognition as alternative treatment strategies as local ablation therapy for HCC treatment (14–17). Among these local ablation therapies, only the efficacy and safety of RFA have been reported in elderly patients with HCC (14, 18–21).

Both RFA and MWA rely on thermal injury, but MWA uses an electromagnetic field as opposed to electrical current used in RFA. Unlike MWA, the effect of RFA is partially limited by the heat-sink effect and increased impedance of the ablated tissue (22). Compared with RFA, MWA attains a more predictable ablation zone, permits simultaneous treatment of multiple lesions, and achieves larger coagulation volumes in a shorter procedural time (23).

Compared to RFA, MWA has a few advantages. First, the heat-sink effect, which occurs when thermal energy from the target lesion is distributed due to blood flow in nearby vessels, is a significant disadvantage of RFA (22). Second, the time required for MWA ablation is smaller than that required for RFA. Third, MWA has the ability to deliver higher temperatures in the ablation zone (24). MWA’s two characteristics result in a more predictable ablation zone (25–27). MWA zones are uniform in shape and size and are not impacted by convective heat loss (24, 28). Because of these benefits, MWA has become a popular therapeutic option for hepatic malignancies. Recently, emerging studies have evaluated the efficacy of MWA of HCC in the elderly with conflicting findings (9, 29–34). Therefore, we conducted this systematic review to assess the safety and feasibility of MWA for HCC in elderly patients.

## Materials and Methods

### Study Design

This study is a systematic review for the critical assessment and evaluation of all published literatures investigating MWA in the elderly population with HCC.

### Ethical Clearance

Ethical clearance for this manuscript was not required because it was a systematic review performed by using prevailing published data.

### Protocol and Registration

This meta-analysis was conducted in accordance with standard guidelines using the Preferred Reporting Items for Systematic Reviews and Meta-Analyses (PRISMA) statement^35^ and has a PROSPERO number CRD42021273091.

### Eligibility Criteria

#### Inclusion Criteria

All studies (controlled or uncontrolled) reporting outcomes of MWA for elderly patients with HCC.

#### Exclusion Criteria

(a) Duplicate studies, case series, case reports, systematic reviews, conference abstracts, preprints, and editorials; (b) studies that do not describe relevant outcomes; and (c) full texts are unavailable.

### Search Strategy

This systematic review was performed following the guidelines of the PRISMA (35) and Cochrane (36). An electronic search of PubMed, Scopus, CENTRAL (Cochrane Central Register of Controlled Trials), and Google Scholar databases was performed for English language papers published up to August 15, 2021. Searches were performed using keywords including “microwave”, OR “microwave ablation”, AND “Elderly”, AND “liver transplantation”, AND “Hepatocellular Carcinoma”, OR “HCC”. Reference lists of the identified studies and relevant reviews on the subject were also scanned for additional studies.

### Data Extraction

Two authors (JZ and HZ) independently extracted the following information from each included study: first author name, country, ethnicity, year of publication, duration of the study, number of patients, treatment methods, study design, duration of the study, group investigated, sample size, number of male/female patients, mean age, cutoff age for elderly definition, tumor size, number of single or multiple tumors, Model For End-Stage Liver Disease (MELD) score, Child–Pugh score, hospital stay duration, objectives, endpoints, and conclusions. Additional information on technical efficacy, local tumor progression, frequency of complications, quality of life, and duration of hospital stay was also extracted from the available included studies. At each stage, publications were examined twice, with conflicts addressed by consensus or adjudication by a third reviewer (DZ).

### Quality Assessment

Assessment of the quality of the included studies was conducted by using the Newcastle–Ottawa Scale (NOS) (37). The NOS comprises the following three aspects: selection of study subjects (4 points), comparability of study subjects (2 points), and exposure or outcomes (3 points). The total score ranges from 0 to 9, and those with a score ≥ 6 were considered as high-quality studies. Two authors independently rated the study’s quality. Any discrepancies in the quality scores were resolved by consensus among the authors.

### Statistical Analysis

Only descriptive analysis of results was performed.

## Results

### Literature Selection

**Figure 1:**
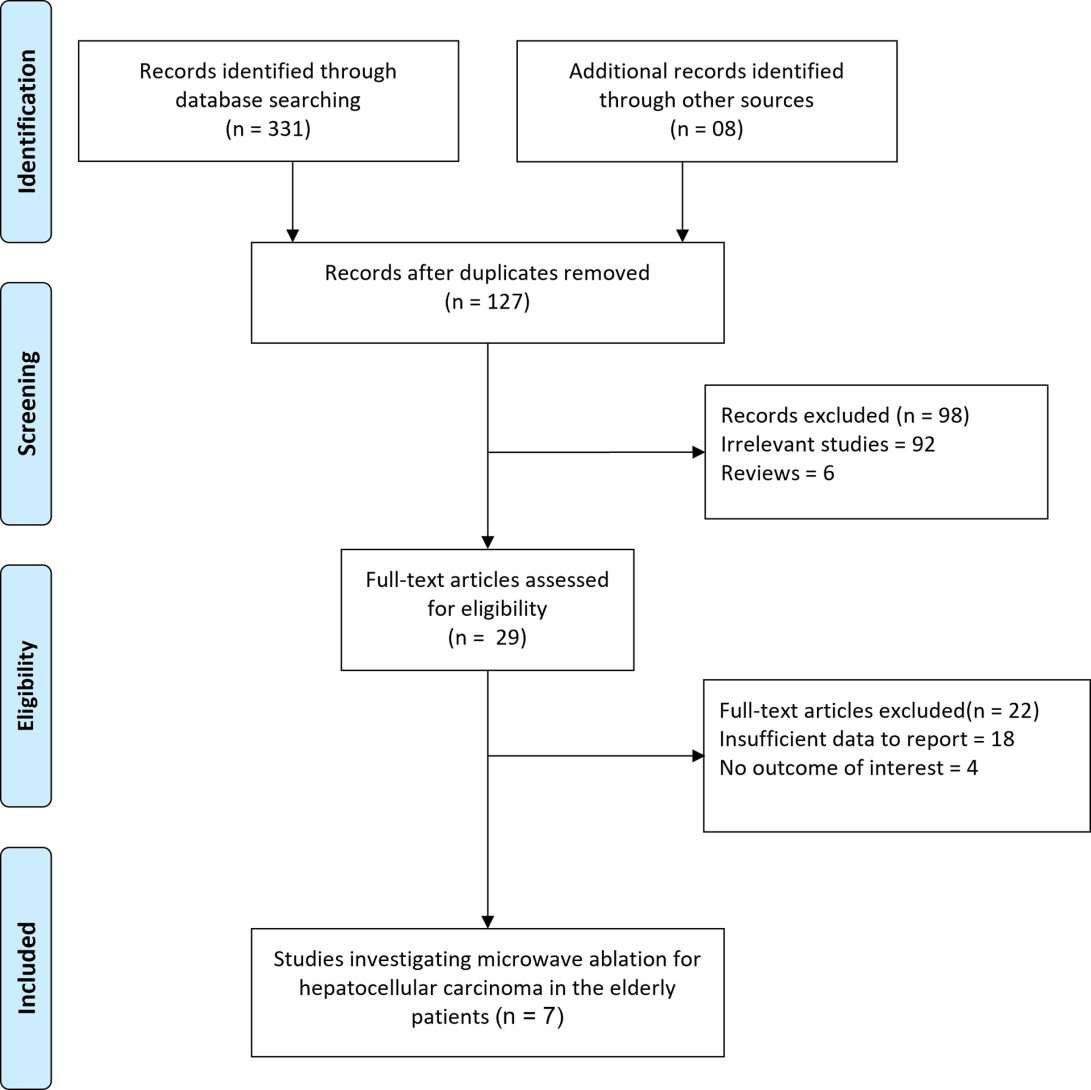
Flow diagram for the selection of studies and specific reasons for exclusion from the present meta-analysis.

The initial search generated 339 records, as shown in **Figure 1**. A total of 127 records were checked after duplicates were removed. After carefully reading the titles and abstracts, 29 articles were selected for further eligibility. Finally, after evaluating full texts, 22 articles were removed due to insufficient data or overlapping data, leaving the current systematic review with 7 total studies.

### Study Characteristics

Seven observational studies (9, 29–34) involving 7,683 HCC patients were included in our systematic review. All included studies were retrospective cohort studies and the published year ranged between 2018 and 2020 with sample sizes of HCC patients ranging from 30 to 2,389. Baseline and clinical characteristics for the included studies are shown in **Tables 1** and **2**. Three studies were conducted in Caucasian patients, while 4 studies were conducted in Asian patients. The median age ranged from 71.4 years to 82.3 years old and median tumor size ranged from 2.4 to 3.2 cm.

**Table 1:** Baseline characteristics of included studies in the systematic review investigating microwave ablation for hepatocellular carcinoma in elderly patients.

S. No.	Author; Year	Country	Study Period	Study Type	Diagnostic Criteria for HCC	Groups	Cutoff Age for the Elderly (years)	Sample Size	M/F	Mean Age	Tumor Size (cm)	No. of Tumors (Single/Multiple)	MELD Score	Child–Pugh score (A/B/C)
1.	Freedman et al., 2020 (29)	Sweden	June 2010–December 2018	R	LIRADS	70–80	70	161	131/30	74.2	NA	88/70	6.2 (0–19.3)	114/18/0
						80–90	80	32	21/11	82.3		20/12	6.5 (0.8–22.7)	24/4/0
2.	Huang et al., 2020 (30)	China	April 2006–October 2019	R	Milan Criteria	Training	65	265	189/76	71.4 ± 5.4	2.8 ± 1.0	192/73		
						Validation		130	87/43	71.4 ± 5.4	2.7 ± 1.0	103/27		
3.	Imamura et al., 2020 (31)	Japan	July 1994–December 2017	R	Pathological examination of a tumor biopsy	NA	80	114	64/50	82	2.6			
4.	Kaibori et al., 2019 (9)	Japan	January 2000–December 2007	R	Milan Criteria	MWA		193						
						RFA		1,888						
						TACE		2,389						
5.	Shen et al., 2018 (34)	China	September 2010–June 2016	R	Chinese Guidelines for the Clinical Diagnosis and Staging of Primary Liver Cancer	<65 years	65	30	23/7					25/5
						≥65 years		35	27/8		2.4	850/203		31/4
6.	Wang et al., 2020 (32)	China	January 2002–December 2017	R	Milan Criteria	<65 years	65	1,053	882/171		2.5	426/84		1015/38
						≥65 years		510	376/134					487/23
7.	Zhang et al., 2020 (33)	China	June 2010–November 2017	R	Chinese Guidelines for the Clinical Diagnosis and Staging of Primary Liver Cancer	<75 years	<75 years	813	670/143	57	2.5		8 (7–9)	783/29/1
						≥75 years	≥75 years	70	46/24	78	3.2		8 (6–8)	68/2/0

LI-RADS, Liver Imaging Reporting and Data System; MELD Score, Model for End-Stage Liver Disease; HCC, hepatocellular carcinoma; MWA, microwave ablation; RFA, radiofrequency ablation; TACE, transarterial chemoembolization; NA, not available; R, retrospective.

**Table 2:** Descriptive summary of findings for the included studies investigating microwave ablation for hepatocellular carcinoma in elderly patients.

S. No.	Author; Year	Groups	Therapies	Follow-up	Objective	Endpoints	Key Findings	Limitations	Conclusion
1.	Freedman et al., 2020 (29)	70–80	MWA	At every 3 months for a year	To evaluate whether it is safe and meaningful to treat octogenarians with MWA for HCC	OS	Octogenarians selected for MWA of HCC at a regional multidisciplinary team conference have similar outcomes to their younger control group. Survival, complications, and length of stay are not different	Small size of the octogenarian cohort could easily mask a Type 2 error. On the other hand, there is the obvious problem with immortality bias as the octogenarians have, by necessity, survived until 80 and are, thus, a selected group with a slightly higher life expectancy. This could perhaps in part explain the excellent 3-year survival of 100% in that cohort	Octogenarians who are fit for ablative treatment of HCC should not be disqualified on grounds of age, recognizing that this group has an obvious immortality, or lead time, bias, as well as a probable selection bias in part explaining their good results.
		80–90							
2.	Huang et al., 2020 (30)	Training	MWA	28.6 months	To develop and validate the nomograms to predict survival outcomes after MWA in elderly HCC patients	OS, RFS	OS nomogram was developed based on HBV presence and albumin, with a C-index of 0.757 (95% confidence interval [CI]: 0.645, 0.789). RFS nomogram was developed based on tumor number, abutting major vessels and platelets, with a C-index of 0.733 (CI: 0.672, 0.774).	Our study has several limitations. First, it was designed as a retrospective study. A prospective cohort study would allow greater elimination of bias in assessing the various risk factors. Second, the long duration of this study may have allowed time for the MWA operators to improve their technique, thereby affecting the rates of ablation efficacy depending on the patient’s time of enrollment in the study.	Nomogram models can be useful in determining the risk of OS and RFS in elderly patients with EHCC after MWA, which can guide individual patient management.
		Validation		24.2 months					
3.	Imamura et al., 2020 (31)		MWA	40 months	To evaluate the feasibility and safety of surgical microwave ablation for HCC in patients older than 80 years of age	OS, RFS	Surgical MWA was feasible and safe for elderly patients with HCC. Elderly patients with HCV-Ab negative and single tumor would be expected to have better long-term outcomes after surgical MWA.	First, it is based on a single-center review and has a limited number of patients. Second, there is the potential for selection bias because of the retrospective design. Lastly, this study did not consider SVR of HCV-Ab-positive patients.	Surgical microwave ablation was feasible and safe for elderly patients with HCC. Elderly patients with HCV-Ab negative and single tumor would be expected to have better long-term outcomes after surgical microwave ablation
	
4.	Kaibori et al., 2019 (9)		MWA RFA TACE		To determine outcomes of different treatments for early-stage HCC in elderly patients.	OS, RFS	MWA was not superior to RFA for RFS and OS. Elderly patients aged >75 years had significantly better RFS after hepatic resection (HR) for HCC than after RFA, MWA, or TACE treatments, and had significantly better OS after HR or RFA for HCC than after TACE treatments.	Lack of data on liver function during the follow-up, which precluded assessment of the relationship between the liver function status and the choice of treatment at recurrence. In HCC, the influence of the initial treatment is considered to be smaller than in other primary malignant diseases because liver function remarkably affects the recurrence rate	HR decreases recurrence risk and improves OS in patients aged 75 years with primary HCC tumors 3.0 cm.
5.	Shen et al., 2018 (34)	<65 years	PMCT	23.5 months	To evaluate the safety and efficacy of ultrasound-guided PMCT in treatment-naive elderly HCC patients, and analyzed risk factors associated with poor treatment outcomes.	Tumor ablation, OS, PFS	Elderly ≥65 age group had a significantly poorer performance status than the <65 age group, but did not differ in other characteristics. Older age was not a predictor of a higher risk of either death or disease progression.	Retrospective nature of the study limits its ability to predict risk factors.	PMCT is safe and effective for patients ≥65 years of age, achieving total ablation in more than 90% of patients. Age and comorbidities did not affect clinical outcome.
		≥65 years							
6.	Wang et al., 2020 (32)	<65 years	MWA		To compare the overall survival (OS), disease−free survival (DFS) and liver−cancer−specific survival (LCSS) of elderly (≥65 years) and younger patients (<65 years) with early−stage hepatocellular carcinoma (HCC) using ultrasound−guided percutaneous microwave ablation (US−PMMA)	OS, DFS, LCSS	No significant differences were detected in OS, DFS, and LCSS between the two groups [elderly (≥65 years) and younger patients (<65 years)]. Complete ablation was achieved in all patients.	(1) A single−center study. A multi−center study should be conducted to confirm the results. (2) Due to its retrospective study design, inherent selection bias could not be eliminated. (3) This study only focused on early−stage HCC patients	Although advanced age and comorbidities are intrinsic factors in elderly HCC patients, similar survival outcomes were obtained in elderly and younger HCC patients treated by US−PMWA, despite elderly patients having more comorbidities.
		≥65 years							
7.	Zhang et al., 2020 (33)	<75 years	MWA		To investigate whether elderly patients with HCC benefit from MWA similar to younger patients.	Prognosis	Elderly patients (aged >75 years) even with a poor comorbidity index benefited from MWA of HCC similar to younger patients with an overall follow-up time of up to 8 years	(1) Retrospective nature. (2) Study was carried out in one center.	Elderly patients with HCC, even though associated with more comorbidities, may achieve acceptable prognostic outcomes following MWA, which are not worse than their younger counterparts.
		≥75 years							

HBsAg, hepatitis B surface antigen; HCVAb, hepatitis C virus antibody; HR, hepatic resection; RFA, radiofrequency ablation; RFS, recurrence-free survival; OS, overall survival; PFS, progression-free survival; PMCT, percutaneous microwave ablation therapy; DFS, disease−free survival; LCSS, liver−cancer−specific survival; HBV, hepatitis B virus; AFP, AFP-a-fetoprotein.

Three studies reported Milan criteria (9, 30, 32), one study used LICRADS (29), one used pathological examination of a tumor biopsy (31), and two studies used Chinese guidelines for the clinical diagnosis and staging of primary liver cancer (33, 34) as the diagnostic criteria for defining HCC. Only two studies (29, 33) reported MELD Score data and four studies (29, 32–34) reported Child–Pugh score data. However, data to determine outcomes of different treatments (MWA/RFA and TACE) for early-stage HCC in elderly patients were only reported by a single study (9). Majority of the studies included were of good quality, with a NOS of six or higher (**Table 3**).

**Table 3:** Quality assessment of the included studies in the systematic review using the Newcastle–Ottawa Scale.

S. No	Study, author, year	Selection				Comparability		Exposure			Quality Score	Quality Grade
		Definition of the non-exposed group	Representativeness of the exposed group	Selection of non-exposed	Definition of non-exposed	Outcome of interest was not present at the start of study	Comparability between the groups	Ascertainment of exposure	Same method of ascertainment for the exposed and non-exposed group	Adequacy of follow-up		
1.	Freedman et al., 2020 (29)	1	1	1	1	0	0	1	0	1	6	Medium
2.	Huang et al., 2020 (30)	1	1	1	1	1	1	1	0	1	8	High
3.	Imamura et al., 2020 (31)	1	1	1	0	1	1	1	0	1	7	High
4.	Kaibori et al., 2019 (9)	1	1	1	1	0	0	1	0	1	6	Medium
5.	Shen et al., 2018 (34)	1	1	1	1	0	0	1	0	1	6	Medium
6.	Wang et al., 2020 (32)	1	1	1	1	0	0	1	0	1	6	Medium
7.	Zhang et al., 2020 (33)	1	1	1	1	1	1	1	0	1	8	High

### Type of Device

Different study protocols were used in all the included seven studies as mentioned briefly in **Table 4** for the type of approach, type of MWA device, type of MW needle, ablation per lesion, average ablation time, and average ablation energy. Only five studies reported the type of MWA device used, namely, MTC-3C (Nanjing, China) (30), Microtaze generator (Alfresa Pharma, Osaka, Japan) (32), FORSEA MTC3C microwave tumor therapy system (Nanjing Qinghai) (35), and KY−2000 (Kangyou Medical, Nanjing, China) [KY-2,00,02,450 MHz (32) and KY-21,00,915 MHz (33)]. The high degree of heterogeneity for the data availability in all the included studies restricted us to perform any analysis in order to reach any conclusive point.

**Table 4:** Detailed MWA characteristics of included studies in the systematic review investigating microwave ablation for hepatocellular carcinoma in elderly patients.

S. No	Author and Year	Type of MWA approach	Type of MWA device	Type of MW needle	Ablation per lesion	Average ablation time	Average ablation energy
1.	Freedman et al., 2020 (29)	CT-guided percutaneous microwave ablation	NR	NR	NR	NR	NR
2.	Huang et al., 2020 (30)	CT-guided percutaneous microwave ablation	MTC-3C, China	20-gauge guided needle	NR	NR	60–70 W
3.	Imamura et al., 2020 (31)	CT-guided percutaneous microwave ablation	Microtaze generator (Alfresa Pharma, Osaka, Japan)	16-gauge 150-mm-long needle or 21-gauge, short needle (range, 10–30 mm)	NR	60 s or 30 s	60 to 65 W or 80 to 85 W
4.	Kaibori et al., 2019 (9)	NR	NR	NR	NR	NR	NR
5.	Shen et al., 2018 (34)	US-guided percutaneous microwave ablation	FORSEA MTC3C microwave tumor therapy system (Nanjing Qinghai)	14G/15 cm microwave antenna	NR	80 W	NR
6.	Wang et al., 2020 (32)	US-guided percutaneous microwave ablation	KY−2000, Kangyou Medical, Nanjing, China	18G cutting needle	NR	50–60 W	NR
7.	Zhang et al., 2020 (33)	US-guided percutaneous microwave ablation	KY-2,00,02,450 MHz and KY-21,00,915 MHz; Kangyou Medical, Nanjing, China	Cool-tip needle antennas of 1.9 mm (15 gauge) in diameter	NR	300 s	60 W

CT, computed tomography; US, ultrasound; NR, not reported.

### Size of Tumor

Kaibori et al. (9) observed that patients over 75 years old with primary HCC with a tumor size of less than 3.0 cm had a lower risk of hepatic resection and had an improved OS. Another study by Shen et al. (34) showed that the elderly (age ≥65 years) group had a considerably poorer performance status than the younger (age <65 years) group, while tumor size and partial ablation were found to be predictors of disease progression. Zhang et al. (33) also found that the size of tumors was a significant predictive variable for OS in a Cox analysis. The tumor size increases with age and may be one of the main reasons for the poorer immune system in elderly patients. Moreover, the probability of liver cancer was higher in those with HCV infection but lower in those with HBV infection as age increased (33).

### Overall Survival

Kaibori et al. (9) suggested that MWA was not superior to RFA for OS. The 5-year OS rates in each group were HR: 59.5%, RFA: 53.2%, MWA: 40.2%, and TACE: 29.2%, and differed significantly among the 4 groups (9). OS was significantly better after HR or RFA for HCC than after TACE treatments in elderly patients aged >75 years. HR: 39.6%, RFA: 34.5%, MWA: 23.8%, and TACE: 19.3%, with significant differences between the four groups. Freedman et al. (29) in their retrospective study comparing first MWA therapy for HCC in septuagenarians (*n* = 161) versus octogenarians (*n* = 32) showed no difference in OS between the two groups, with a median survival time of 3.9 years for patients between 70 and 80 years of age and 4.3 years for octogenarians (*p* = 0.416). The older group had an average age of 82 and a median survival of 4.3 years, whereas the younger cohort had an average age of 74 and a median survival of 3.9 years.

Another finding by Wang et al. (32) showed no significant differences in OS between two groups [elderly (more than 65 years) and younger patients (less than 65 years)]. HCV infection, comorbidities, cirrhosis, larger tumors, poor liver functional status, more ablation points, longer ablation time, longer hospital stays, and greater hospitalization expenditures were all more common in elderly individuals. Albumin, r-glutamyl transpeptidase (rGT), and ablation session were found to be significant predictors for OS.

Huang et al. (30) developed and validated nomograms to predict survival outcomes after MWA in 265 early-stage HCC (EHCC) patients showed that older patients with EHCC who had MWA had satisfactory OS rates, with a 10-year rate of 32.8%. Multiple tumors, abutting major vessels, and low platelet levels were related with significant recurrence rates following MWA; HCV or other etiologies, high AFP levels, and low albumin levels were associated with a low OS rate. They concluded that that OS in patients over 75 years old was equivalent to that in individuals 65 to 75 years of age.

Imamura et al. (31) also found that surgical MWA can be performed safely and effectively in older patients with primary HCC, with a 5-year OS rate of 49.2%. HCV-Ab positivity and multiple tumors were found to be independent predictive variables for OS in their multivariate analysis. Zhang et al. (33) also confirmed that elderly patients (age >75 years), even with a poor comorbidity index, benefited from MWA of HCC similar to younger patients with an overall follow-up time of up to 8 years. After matching, there were no significant differences in the rates of complete ablation and major complications, as well as OS and PFS, between those aged >75 years and those aged <75 years. The findings of Shen et al. (34) also suggested that older age was not associated with an increased risk of mortality or disease progression. Multiple tumors, hypertension, and lower postoperative ALT levels were found to be predictors of death. Their data imply that there is no link between age and clinical success following HCC treatment with percutaneous microwave ablation therapy (PMCT) (34).

## Discussion

The prevalence of HCC in elderly people is expected to continue to increase in the near future (3, 38). Minimally invasive therapy is often recommended in elderly patients considering their reduced tolerance to surgery and the presence of comorbidities. In recent years, interest in MWA has increased due to its potential physical advantages, which have been facilitated by modern high-powered devices (39). Microwaves may provide more direct heating than other energies, making MWA more effective in organs with high blood perfusion or near vascular heat sinks than other thermo-ablative modalities. A previous systematic review and meta-analysis conducted by Glassberg et al. (40) indicated that MWA is safe and effective as RFA for the treatment of HCC or liver metastases and MWA is significantly associated with lowering the rates of local tumor progression as compared to RFA. MWA obtains a larger area of tumor necrosis compared with RFA. At present, MWA with a water-cooling cycle can obtain a larger ablation boundary and avoid the effect of tissue electrical conduction, and tissue carbonization prevents the effect of its energy diffusion (41, 42).

To our knowledge, this is the first systematic review to qualitatively show that elderly patients, despite having a high comorbidity index, benefited from MWA of HCC in a similar manner to younger patients. It should be noted that all included clinical studies are retrospective (9, 29–34). This increases the risk of clinical consequences being under-reported or misreported. Furthermore, in all investigations that included MWA, no severe problems were reported. Even with a high comorbidity index, elderly patients benefited from MWA of HCC in a similar manner to younger patients with a longer overall follow-up time (9, 29–34). Although advanced age and comorbidities are fundamental variables in older HCC patients, senior and younger HCC patients treated by ultrasound percutaneous MWA had similar survival outcomes, despite elderly patients having greater comorbidities (32). However, most of the included studies in our review confirmed that MWA can be performed safely and effectively in older patients with primary HCC with a similar overall survival to younger subjects.

Shen et al. (34) confirm that older age was not associated with an increased risk of mortality or disease progression. Multiple tumors, hypertension, and lower postoperative ALT levels were found to be predictors of death, while tumor size and partial ablation were found to be predictors of disease progression. Huang et al. (30) developed a clinicopathological-based nomogram having the consistent ability to predict survival outcomes in elderly with HCC and showed that multiple tumors, abutting major vessels, and low platelet levels were related with significant recurrence rates following MWA; HCV or other etiologies, high AFP levels, and low albumin levels were associated with a low OS rate. Zhang et al. also suggested that the size of tumors and Child–Pugh grade, rather than age or the Charlson comorbidity index, were found to be significant predictive variables for OS in a Cox analysis (33). Therefore, summarizing lines of evidence suggests that age and comorbidities may not have an effect on MWA in older HCC patients, which could assist in broadening the criteria for MWA in clinical practice.

There were some limitations in our systematic review: (1) only limited number of studies were published investigating the impact of MWA in the elderly; (2) all the included studies (*n* = 7) were of retrospective nature, which may lead to recall bias for the observed findings; (3) we could not perform a meta-analysis due to the availability of heterogenous data in all the included studies; (4) findings must be interpreted with caution as the definition for elderly age varied in the included studies; (5) different types of available MWA machines may have varying efficacies and were not reported; (6) tumor numbers (single/multiple) varied in the elderly and only few articles reported tumor size; and, lastly, (7) all included studies were conducted over different time periods and an increase in the experience of operators may affect results.

## Data Availability Statement

The original contributions presented in the study are included in the article. Further inquiries can be directed to the corresponding author.

## Author Contributions

WL and WH conceived and designed the study. JZ, HZ, DZ, and HY collected the data and performed the analysis. GS was involved in the writing of the manuscript and is responsible for the integrity of the study. All authors contributed to the article and approved the submitted version.

## Funding

This work was supported by the Medical and Health Science and Technology Plan Project in Zhejiang Province of China (No. 2021427572).

## Conflict of Interest

The authors declare that the research was conducted in the absence of any commercial or financial relationships that could be construed as a potential conflict of interest.

## Publisher’s Note

All claims expressed in this article are solely those of the authors and do not necessarily represent those of their affiliated organizations, or those of the publisher, the editors and the reviewers. Any product that may be evaluated in this article, or claim that may be made by its manufacturer, is not guaranteed or endorsed by the publisher.
